# Fabrication of Micro-Structured Polymer by Micro Injection Molding Based on Precise Micro-Ground Mold Core

**DOI:** 10.3390/mi10040253

**Published:** 2019-04-16

**Authors:** Yanjun Lu, Fumin Chen, Xiaoyu Wu, Chaolan Zhou, Yan Lou, Liejun Li

**Affiliations:** 1 Guangdong Provincial Key Laboratory of Micro/Nano Optomechatronics Engineering, College of Mechatronics and Control Engineering, Shenzhen University, Shenzhen 518060, China; luyanjun@szu.edu.cn (Y.L.); 2172291706@email.szu.edu.cn (F.C.); wuxy@szu.edu.cn (X.W.); susanlou121@163.com (Y.L.); 2 School of Mechanical and Automotive Engineering, South China University of Technology, Guangzhou 510640, China

**Keywords:** micro-grinding, micro-structure, micro injection molding, filling rate, polymer

## Abstract

Precise micro-grinding machining was proposed to fabricate regular and controllable micro-grooved array structures on the surface of mold cores to realize the mass production and manufacturing of micro-structured polymer components by micro injection molding in this paper. First, the 3D topographies and section profiles of micro-ground mold cores and micro-formed polymers with different micro-structure parameters were presented. Then, the surface roughness of mold cores and polymers were compared. Next, the relationships between machining accuracy of mold core ground by micro-grinding and filling rates of micro-structured polymer formed by micro injection molding were investigated. Finally, the influences of micro injection molding parameters on the filling rate of micro-structures polymer were investigated. It is shown that the micro-structured polymer can be effectively and rapidly fabricated using the proposed method. The experimental results indicate the highest form accuracy of the micro-grooved mold core and the filling rate of micro-structured polymer can reach to 4.05 µm and 99.30%, respectively. It is found that the filling rate of the micro-structured polymer roughly increased with increasing machining accuracy of the mold core. The injection pressure had the greatest influence on the filling rate of the injection formed polymer, while the melt temperature had the least influence.

## 1. Introduction

Micro-structured polymer components have been widely used in many fields, such as optics, biomedicine, electron and microelectro-mechanical systems (MEMS), etc. [1,2,3]. At present, micro-forming technologies of micro-structured polymer mainly include micro injection molding, micro hot embossing molding, and injection compression molding, and so on [4,5]. It had been confirmed that the micro injection molding was an alternative to the mass production and manufacturing of micro-structured polymer products due to its short molding cycle, high efficiency, and low production cost [6,7]. For instance, the superhydrophobic polymer surfaces with hierarchical micro-nano cylinder array structures were successfully fabricated by micro injection molding [8]. It was shown that the contact angle of the droplet on the hierarchical micro-nano structured polymer surface reached about 163°, resulting in the self-cleaning characteristic. An amorphous polymer surface with high aspect ratio micro-structures had been fabricated by micro injection molding for application in bio-MEMS for erythrocytes depletion [9]. It had been verified that the mold temperature had the largest influence on the replication degree of micro-features against other main process parameters. The micro injection molding was employed to manufacture a micro-pillared polymer surface by replicating the micro features from the mold insert with micro-cavity structures [10]. The experimental results showed that small cavity thickness and high molding temperature had a positive effect on the replicated height of micro-features.

However, it is very difficult to realize high form accuracy machining and manufacturing of micro-nano structures on the surface of mold core, which directly determined the micro-forming quality of micro-structured polymer products. For micro-nano scale machining of the micro-structured mold core surfaces, there were many advanced processing technologies, such as chemical etching [11], laser processing [12], electrical discharge machining (EDM) [13], fluid jet-array parallel machining (FJAPM) [14], etc. Although chemical etching and laser processing technologies can fabricate micro-textured structures at the nanoscale, they were very difficult to use to ensure the 3D form accuracy of micro-structures at the micron scale. In spite of the fact that the electrical discharge machining (EDM) could efficiently fabricate complicated 3D micro-structures, it was very difficult to reach the smooth micro-structured surface with. The fluid jet-array parallel machining (FJAPM) may machine smooth micro-structured surfaces, but it needed to take a lot of processing time. It was found that a dressed super-abrasive diamond grinding wheel may perform precision micro-grinding for mold core to obtain smooth micro-structured surface [15,16,17]. In order to achieve high form accuracy and surface quality of micro-structures at the micron scale, the efficient and precise micro-grinding technology was proposed to fabricate micro-grooved array structures with controllable form accuracy on the surfaces of the mold core in this work. Moreover, the operation process of micro-grinding machining was very simple, and the manufacturing cost was relatively low.

In this paper, the regular and controlled micro-grooved array structures on the surface of mold core were machined by micro-grinding machining using a trued V-tip diamond grinding wheel. The micro injection molding technology was employed to rapidly fabricate micro-structured polymer parts by replicating the micro-features of mold core surface. The surface topographies and V-groove profiles of micro-structured mold cores and polymers were presented to analyze the form accuracy of micro-grinding and filling rate of micro injection molding. The surface roughness of micro-ground mold cores and micro-formed polymers were compared. The relationships between the form accuracy of micro-ground mold core and filling rates of micro-structured polymers were also revealed. The influences of micro injection molding parameters on the filling rate of micro-structures polymer were also analyzed.

## 2. Materials and Methods

### 2.1. Micro-Grinding of Mold Core with V-Grooved Array Structures

Compared with the traditional mold core materials, Titanium silicon carbide (Ti_3_SiC_2_) exhibited both metal and ceramic properties, including excellent machinability, good electrical conductivity, high wear resistance, and good self-lubrication, etc. [18,19]. In this experiment, Ti_3_SiC_2_ ceramic was chosen as mold core material due to its good lubrication and demolding performances.

**Figure 1:**
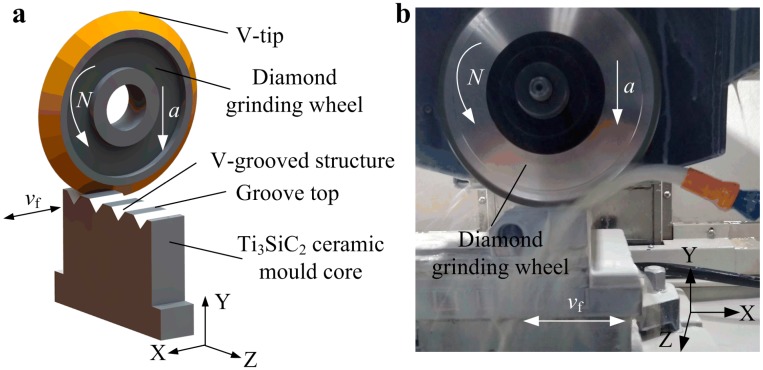
Micro-grinding machining scheme and photo of mold core: (**a**) Schematic diagram of V-grooved structures machining; (**b**) Photo of micro-grinding.

Figure 1 shows the micro-grinding machining scheme and photo of Ti_3_SiC_2_ mold core surface with V-grooved array structures through a computer numerical control (CNC) precision grinding machine. First, a V-shaped tip of diamond grinding wheel was trued through mechanical V-tip mutual truing technology along CNC interpolation trajectory [20]. Then, the Ti_3_SiC_2_ ceramic mold core was mounted on the horizontal work table of the grinding machine. The trued V-tip diamond grinding wheel was driven to grind the Ti_3_SiC_2_ mold core through numerical control system (see Figure 1a). The V-groove was gradually formed on the surface of Ti_3_SiC_2_ mold core through replicating the V-tip shape of the diamond grinding wheel. When a V-groove was completed, the diamond grinding wheel was moved at a setting space along the Z-axis direction to perform the grinding machining of second V-groove. Finally, the micro-grooved array structures were obtained on the surface of mold core according to the setting machining path. The machining photo of the mold core was shown in Figure 1b. Because the Ti_3_SiC_2_ belongs to the ceramic material, according to previous machining experiences, the detail micro-grinding conditions of mold core using V-tip diamond grinding wheel were chosen and shown in Table 1. Under the same grinding conditions, the designed six sets of V-grooved structures were ground on the surfaces of mold cores. The designed V-grooved parameters included V-groove angle *α*, V-groove depth *h* and V-groove space *b* which were shown in Table 2. The corresponding mold cores with different V-grooved array structures were labeled as Sample A_m_, B_m_, C_m_, D_m_, E_m_ and F_m_, respectively.

### 2.2. Micro Injection Molding of Micro-Structured Polymers

**Figure 2:**
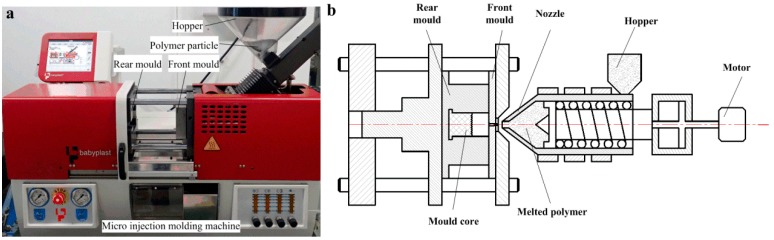
Photo and working principle of the micro injection molding machine: (**a**) photo; (**b**) working principle.

The V-grooved array structures of mold core surface can be replicated on the surface of workpieces through a micro injection molding machine (Babyplast 6/10P, Cronoplast Sl, Barcelona, Spain) as shown in Figure 2a. The highly efficient and accurate micro injection molding machine was very suitable for mass production and processing of all thermoplastic micro precision parts due to its metal ball plasticizing system and piston injection system. Figure 2b shows the working principle of the micro injection molding machine. The polypropylene (PP) particles (B310, Lotte Chemical Corporation, Seoul, Korea) were chosen as polymer workpiece materials and were placed in a hopper in this experiment. The flow rate, density, thermal deformation temperature and melting point of polymer material were 0.5 g/10 min, 0.9 g/cm^3^, 110 °C and 167 °C, respectively. The mold core with V-grooved array structures was installed in the rear mold. The polymer particles were first heated and plasticized, then were melted and injected into a cavity of front mold through the nozzle under the action of plunger driven by the motor. Next, the cavity would be cooled after holding pressure for a certain time. Finally, the micro-structured polymer was formed when the front and rear molds were simultaneously released.

**Figure 3:**
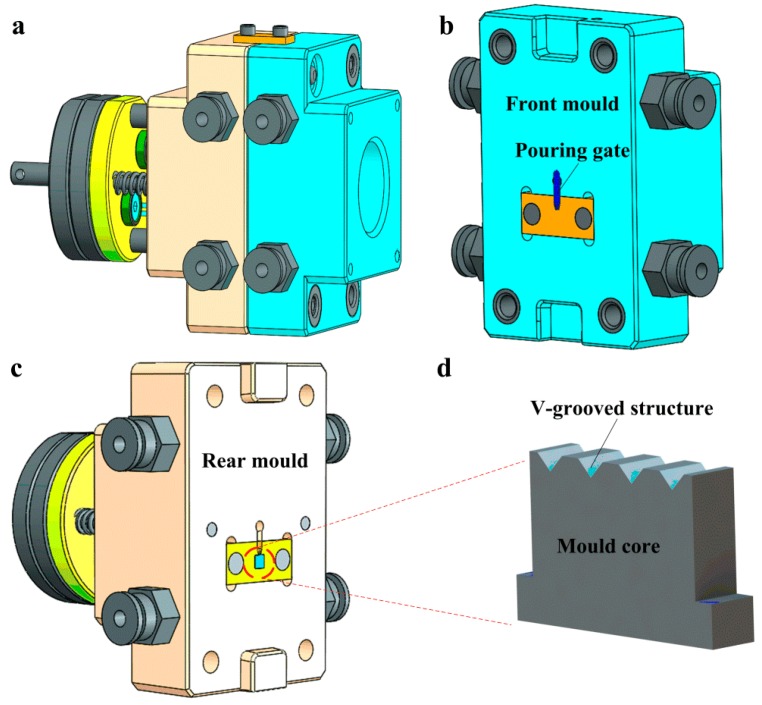
The design sketches of mold frame and mold core: (**a**) the whole mold frame; (**b**) the front mold; (**c**) the rear mold; (**d**) the mold core.

Figure 3 shows the design sketches of the mold frame and mold core. The structural diagram of the whole mold frame was shown in Figure 3a. Figure 3b shows the schematic diagram of the front mold. The melted and plasticized polymer materials were injected into the cavity of the front mold through the pouring gate. Figure 3c shows the schematic diagram of the rear mold installed with the mold core. Figure 3d shows the schematic diagram of the mold core with V-grooved structures ground by micro-grinding technology.

**Figure 4:**
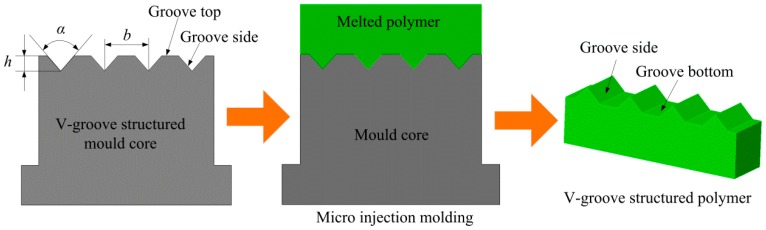
Principle of micro injection molding of the micro-structured polymer.

Figure 4 shows the micro injection molding process of micro-structured polymer. After the micro injection molding, the V-groove of the mold core was duplicated on the surface of the polymer to form an inverted V-shaped groove structure. The V-groove parameters of micro-ground mold core were described as V-groove angle *α*_1_, V-groove depth *h*_1_ and V-groove space *b*_1_. The V-groove parameters of micro-structured polymer were described as V-groove angle *α*_2_, V-groove depth *h*_2_ and V-groove space *b*_2_. The surface qualities of micro-structured polymer at groove side and groove bottom depend on the groove side and groove top of mold core, respectively. The six groups of micro-ground mold cores were employed to conduct micro injection molding experiments under the same conditions. According to preliminary experimental experiences, the melting temperature, injection speed, holding pressure and holding time was set as 210 °C, 40 mm/s, 7 MPa and 5 s, respectively. After micro injection molding, the corresponding micro-structured polymer samples were defined as A_w_, B_w_, C_w_, D_w_, E_w_ and F_w_, respectively.

In order to investigate the influences of melt temperature *T*, injection speed *v*, injection pressure *P* and holding time *t* on filling rate of micro-structures polymer, the experimental parameters were designed and presented in Table 3. Under each process parameter condition, thirty micro-molding polymer samples were produced, and five samples were randomly selected for testing and averaging.

### 2.3. Measurement of Micro-Grooved Mold Cores and Polymers

High-resolution scanning electron microscopy (SEM, FEI Quanta 450FEG and Apreo S, FEI Company, Hillsboro, OR, USA) was used to detect the surface topographies of micro-structured mold cores. The 3D laser scanning microscope (VK-250, Keyence, Osaka, Japan) was employed to measure the 3D topographies and section profiles of micro-structured mold cores. A probe stepper (D-300, KLA-Tencor, Milpitas, CA, USA) was employed to measure the section profiles of micro-structured polymers. Through the section profiles curve, the surface roughness and V-tip angle can be obtained through the data analysis software. The presented result was the average value of five measured data.

## 3. Results and Discussions

### 3.1. Surface Topographies and Profiles of Micro-Ground Mold Core

**Figure 5:**
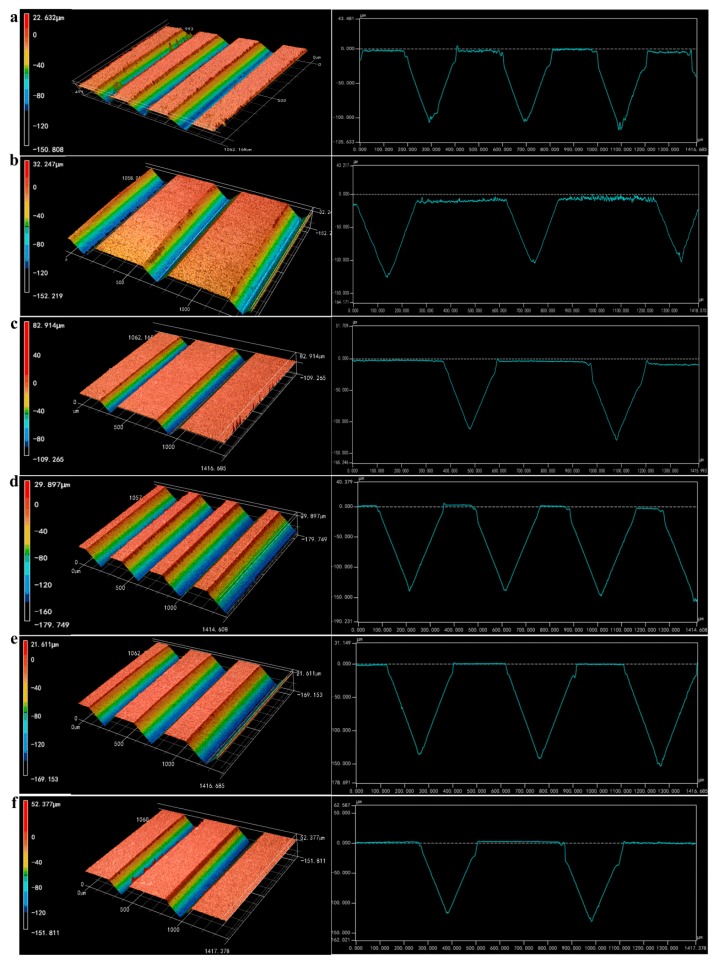
3D topographies and profiles of micro-ground mold cores: (**a**) Sample A_m_; (**b**) Sample B_m_; (**c**) Sample C_m_; (**d**) Sample D_m_; (**e**) Sample E_m_; (**f**) Sample F_m_.

Figure 5 shows the 3D topographies and section profiles of the mold core with micro-grooved array structures after micro-grinding. It is shown that the regular and smooth V-grooved array structures were integrally fabricated on the surfaces of mold cores. The micro-ground V-grooved structure parameters were shown in Table 4, which were roughly consistent with designed V-grooved parameters shown in Table 2. The V-tip angle *α* was derived from the V-groove profile curve measured by the 3D laser scanning microscope. The micro-ground angle errors of V-groove were ranged from 0.88°–1.87° and the average angle error was 1.38°. The micro-ground average depth and space errors of V-groove were 2.62 µm and 2.73 µm, respectively. The actual V-groove space of mold core was slightly larger than the theoretical value, which was mainly affected by the end face jump of the diamond wheel.

**Figure 6:**
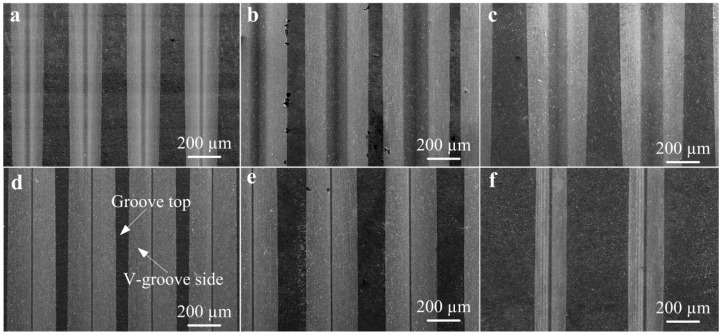
SEM photos of micro-ground mold cores: (**a**) Sample A_m_; (**b**) Sample B_m_; (**c**) Sample C_m_; (**d**) Sample D_m_; (**e**) Sample E_m_; (**f**) Sample F_m_.

Figure 6 shows the SEM photos of micro-ground mold core surfaces. As seen from the SEM observations, the surface topography characteristics of the V-groove were substantially identical to measured 3D topographies shown in Figure 5. It is also seen that the surface of V-groove side was smoother than the one of groove top (un-ground surface). The Sample D_m_ had the smoothest and most regular V-groove surface compared with the other Samples.

### 3.2. Surface Topographies and Profiles of Micro-Structured Polymers

**Figure 7:**
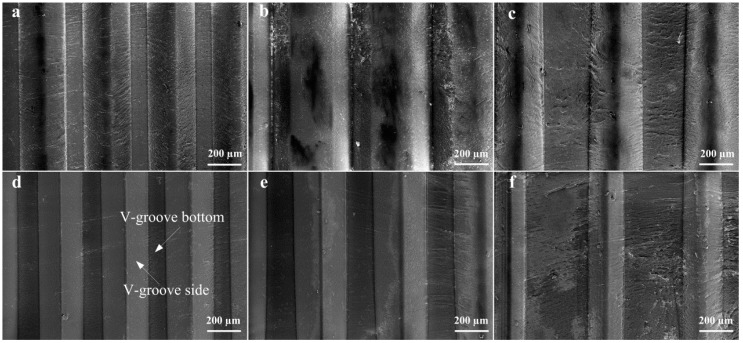
SEM photos of micro-structured polymers: (**a**) Sample A_w_; (**b**) Sample B_w_; (**c**) Sample C_w_; (**d**) Sample D_w_; (**e**) Sample E_w_; (**f**) Sample F_w_.

Figure 7 shows the SEM topographies of micro-structured polymers after micro injection molding. As seen from the SEM photos, the V-shaped groove array structures of mold cores were preferably replicated on polymers to form an inverted V-shaped groove structure. It is shown that the micro-structured surface of D_w_ was the smoothest and the surface roughness of groove side *R*_a_ reached 0.052 μm. Moreover, it is seen that the V-groove side of the micro injection molded polymer was smoother than the one of the mold core (see Figure 6d and Figure 7d). It is observed that there were many cracks and melted polymers on the micro-formed surfaces for Samples B_w_, C_w_, and F_w_. The reason is that the micro-ground surface qualities of corresponding mold cores were not satisfactory, leading to difficult demolding in the micro injection molding process. For all micro-formed polymers, the V-groove side was smoother than the V-groove bottom. This is because the surface quality of groove bottom of polymers depended on the un-ground groove top of mold cores.

**Figure 8:**
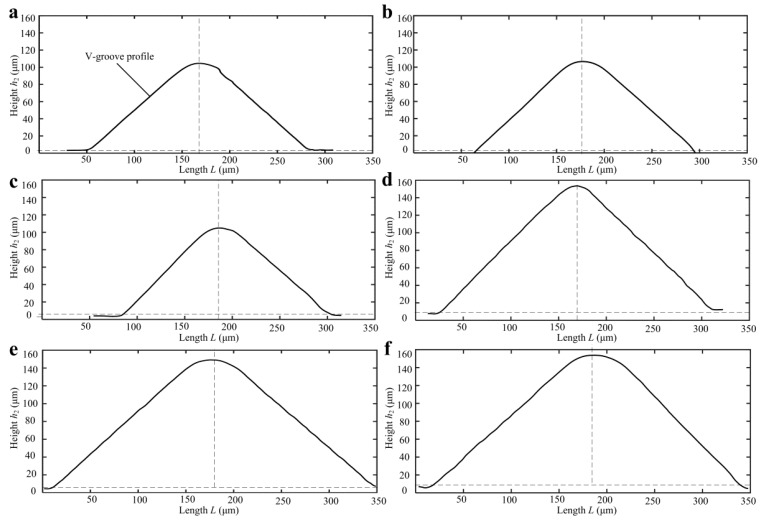
The profiles of micro-structured polymers: (**a**) Sample A_w_; (**b**) Sample B_w_; (**c**) Sample C_w_; (**d**) Sample D_w_; (**e**) Sample E_w_; (**f**) Sample F_w_.

Because the polymer was nonopaque, the cross-sectional profiles of micro-structured polymer surfaces were captured by a contact step profiler. Figure 8 shows the V-grooved section profile curves of micro-structured polymers after micro injection molding. The V-grooved structure parameters of polymers are shown in Table 5. The V-tip profile curve had an arc radius due to the V-tip arc radius of the mold core and trued diamond grinding wheel. Compared with designed V-grooved parameters shown in Table 2, the micro-formed angle errors of V-groove on the surface of micro-structured polymers were ranged from 0.02°–0.88° and the average angle error was only 0.46°. The micro-formed average depth and space errors of V-groove were 2.42 µm and 1.12 µm, respectively. Compared with the V-tip profiles, it is found that Sample D_w_ had the highest micro-forming accuracy. The results were also consistent with the SEM photographs shown in Figure 7.

### 3.3. Machining Accuracy of Micro-Ground Mold Core and Filling Rate of Micro-Formed Polymer

Although the measurement instruments of mold core and polymer profiles are different, based on previous measurement experiences, the measurement results of the two instruments are almost the same. Through comparing with the V-groove profiles of mold cores and micro-tip of grinding wheel tool, the profile error distribution and angle error curve can be obtained. The profile error *e*_m_ was defined as the height deviation between the V-groove profile of mold core and the micro tip profile of diamond grinding wheel. The V-groove angle relative error of mold core α_m_ can be calculated by the following formula:

(1) $$\alpha_{m}=\frac{\left|\alpha -\alpha_{1}\right|}{\alpha }\times 100\%$$

where *α* is the V-groove angle of the wheel tool tip; *α*_1_ is the V-groove angle of micro-structured mold core. Because of the misalignment of the end face of the diamond grinding wheel, the V-tip angle of mold core surface was generally larger than the one of the wheel tool tip. The form accuracy of mold core *γ* can be defined as the difference value between a peak and a valley at the profile error curve [21].

**Figure 9:**
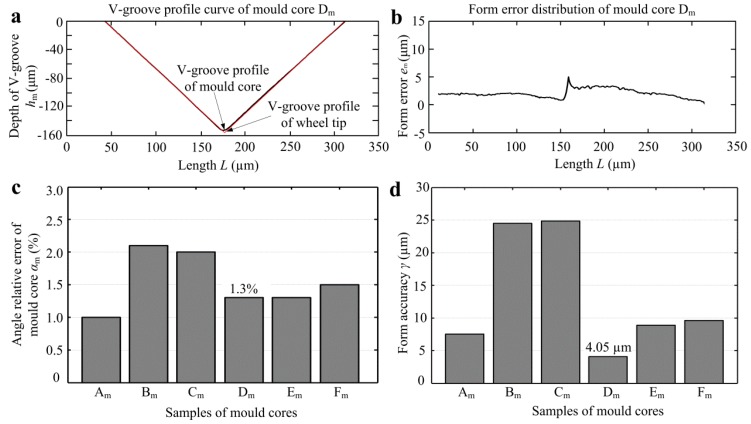
The angle relative error *α*_m_ and form accuracy *γ* of mold core: (**a**) V-groove profile curves of wheel tip and mold core D_m_; (**b**) form error distribution of mold core D_m_; (**c**) angle relative error *α*_m_; (**d**) form accuracy *γ*.

Figure 9 shows the angle relative error *α*_m_ and form accuracy *γ* of mold cores. According to the V-groove profile curves of mold core and wheel tool tip as shown in Figure 9a, the form error distribution curve of mold core can be obtained as shown in Figure 9b. It indicated that the maximum profile error emerged at the tip of V-groove. This is because the micro machining of V-shaped tips has always been a technical bottleneck so that the arc radius would always be existent. It is shown that V-tip profile error of micro-ground mold core can be controlled within 5 µm. Figure 9c shows the angle relative errors *α*_m_ of all mold cores, which were ranged from 1.0%–2.1%. The average angle relative error of micro-ground mold cores was only 1.53%. Through the form error distribution curve shown in Figure 9b, the form accuracy *γ* can be obtained as shown in Figure 9d. It is shown that mold core D_m_ had the minimum form accuracy of 4.05 µm. Besides mold cores B_m_ and C_m_, the micro-ground form accuracies of other mold cores were below 10 µm.

Through comparing with the V-groove profiles of mold cores and polymers, the profile error distribution and angle error can be obtained. Therefore, the V-groove angel relative error of polymer *α*_w_ can be computed as follows:

(2) $$\alpha_{w}=\frac{\left|\alpha_{1}-\alpha_{2}\right|}{\alpha_{1}}\times 100\%$$

where *α*_2_ is the V-groove angle of micro-structured polymer. The filling rate of micro injection molding *η* can be obtained by the following equation:

(3) $$\eta =1-\frac{1}{N}\sum \frac{\left|h_{1}-h_{2}\right|}{h_{1}}\times 100\%$$

where *h*_1_ is the V-groove depth value of mold core, *h*_2_ is the V-groove depth value of polymer workpiece, *N* is the data points of the measured V-groove profile.

**Figure 10:**
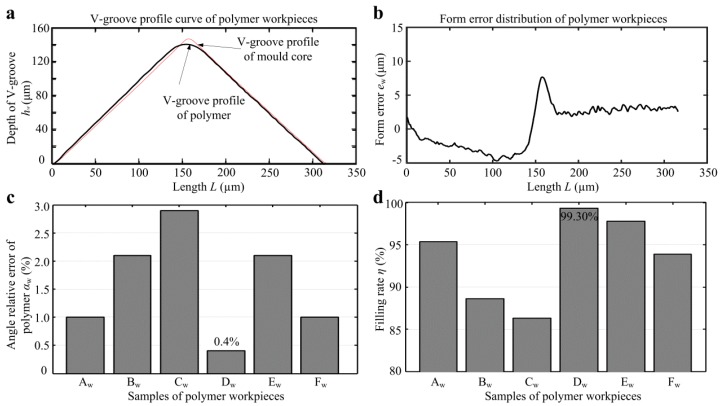
The angle relative error *α*_w_ and filling rate *η* of micro-structured polymer: (**a**) V-groove profile curves of polymer D_w_ and mold core D_m_; (**b**) form error distribution of polymer D_w_; (**c**) angle relative errors *α*_w_; (**d**) filling rates *η*.

Figure 10 shows the angle relative error *α*_w_ and filling rate *η* of micro-structured polymers. Figure 10c shows the angle relative error *α*_w_ for all micro-structured polymers, which were ranged from 0.4%–2.9%. The average angle relative error of micro-formed polymers was only 1.58%. It is shown that polymer D_w_ had the minimum angle relative error of 0.4%. It is also found that the V-tip angles of micro-structured polymers were less than the ones of V-grooved mold cores. This is because the injection molded polypropylene (PP) had shrinkage during the cooling process, resulting in a decrease in the V-groove angle. As observed from Figure 10c, the larger the space of V-groove was, the larger the angle relative error was. The reason may be that the larger the space of V-groove was, the faster the shrinkage of the micro-formed polymer was, resulting in an increase in V-groove angle. Figure 10d shows the filling rate *η* for all micro-structured polymers. It is seen that the workpiece D_w_ had the highest filling rate, which reached up to 99.30%. Through comparing with Figure 9d and Figure 10d, it can be basically concluded that the higher the form accuracy of mold core machining was, the higher the filling rate of the micro-formed polymer was. Besides, it is also found that the larger the depth or the depth-width ratio of V-groove was, the higher the filling rate was.

### 3.4. Surface Quality Analysis of Mold Core and Injection Molded Polymers

**Figure 11:**
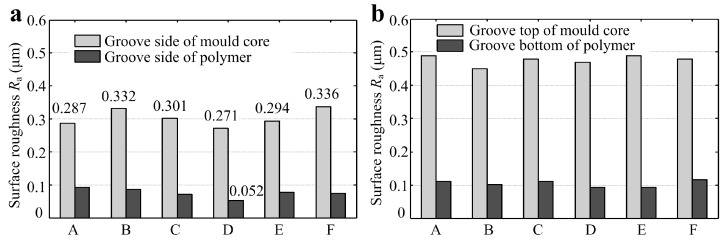
Comparisons of surface roughness *R*_a_ of micro-structured mold cores and polymers: (**a**) groove sides of mold core and polymer; (**b**) groove top of mold core and groove bottom of the polymer.

According to the micro-forming principle of micro-structured polymer fabricated by micro injection molding as shown in Figure 4, the surface qualities of the polymer at groove side and groove bottom depend on the micro-ground groove side and un-ground groove top of mold core, respectively. Figure 11a shows the surface roughness *R*_a_ of groove sides of mold cores and polymers. The comparisons of surface roughness *R*_a_ between the groove top of mold cores and groove bottom of polymers were shown in Figure 11b. It can be observed that the surface roughness *R*_a_ of groove sides of micro-ground mold cores and micro-formed polymers were ranged from 0.271–0.336 μm and 0.052–0.092 μm, respectively. This indicated that the surface qualities of injection molded polymers were better than the ones of mold cores. The groove side of micro-ground mold core D_m_ had the least surface roughness *R*_a_ of 0.271 μm and the corresponding surface roughness *R*_a_ of polymer D_w_ also achieved a minimum value of 0.052 μm. The surface roughness *R*_a_ of the groove side of micro-formed polymers can be maintained below 0.1 μm. It is also shown that the un-ground groove top of mold cores was much rougher than the micro-ground groove side, leading to degradation in the surface quality of groove bottom of micro-structured polymers.

### 3.5. Relationship between Filling Rate of Micro-Structured Polymer and form Accuracy of Micro-Ground Mold Core

**Figure 12:**
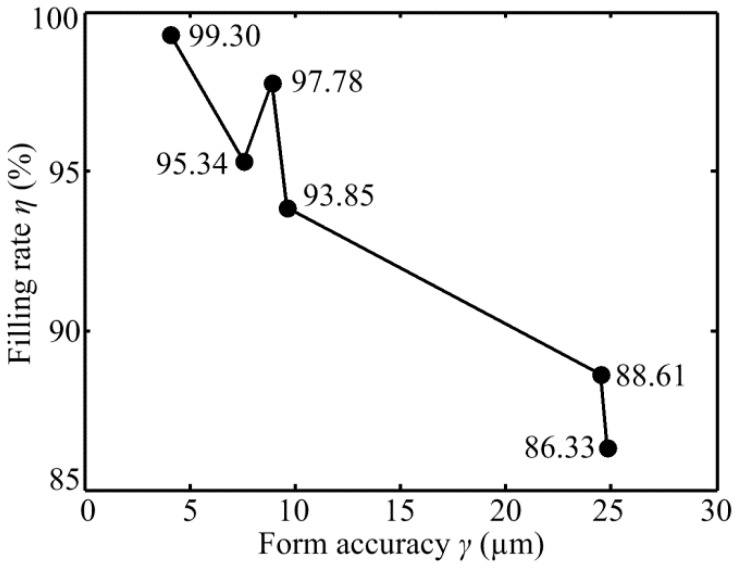
The relationship between the filling rate of micro-structured polymer *η* and form accuracy of micro-ground mold core *γ*.

Figure 12 shows the relationship between the filling rate of micro-structured polymer *η* and form accuracy of micro-ground mold core *γ*. The micro-ground form accuracies of six mold cores were 7.53 µm, 24.5 µm, 24.84 µm, 4.05 µm, 8.87 µm, and 9.59 µm, respectively. The corresponding filling rate of micro-formed polymers were 86.33%, 95.34%, 88.61%, 93.85%, 97.78% and 99.45%, respectively. It is shown that the form accuracy of micro-ground mold core had a positively influences on filling rate of micro injection molding. The filling rate of micro-structured polymer basically increased with the decrease of the form accuracy of micro mold core. This indicated that the higher machining accuracy of the mold core was, the higher the filling rate of micro injection molding was.

### 3.6. Effects of Micro Injection Molding Parameters on the Filling Rate of Micro-Structured Polymer

**Figure 13:**
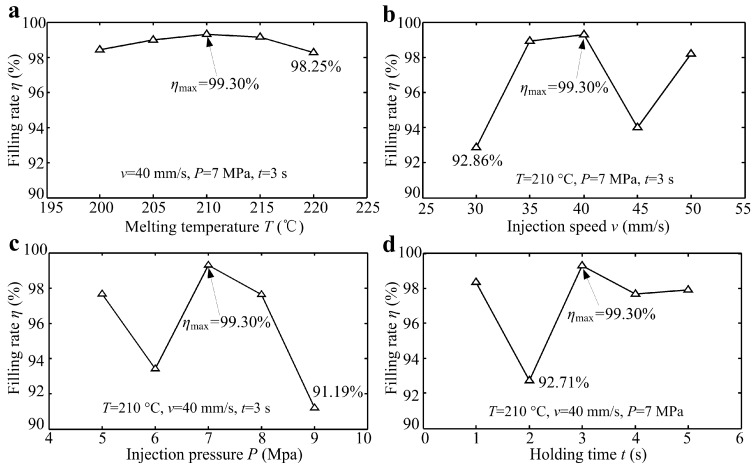
The filling rate of micro-structured polymer *η* versus micro injection molding parameters: (**a**) Melting temperature *T*; (**b**) injection speed *v*; (**c**) injection pressure *P*; (**d**) holding time *t*.

Figure 13 shows the filling rate of micro-structured polymer η versus micro injection molding parameters including melting temperature T, injection speed v, injection pressure P and holding time t. As seen from Figure 10a,b, the filling rate η of micro-formed polymer basically first increased and then decreased with the increase of melt temperature T and injection speed v. The filling rate of micro-formed polymer was ranged from 98.25%–99.30% and 92.86%–99.30%, respectively. According to the results shown in Figure 10c,d, the change range of filling rate with injection pressure P and holding time t was 91.19%–99.30% and 92.71%–99.30%, respectively. Therefore, the experimental results showed that the injection pressure had the greatest influence on filling rate of the injection formed polymer, while the melt temperature had the least influence. In summary, when the melting temperature, injection speed, injection pressure and holding time was 210 °C, 40 mm/s, 7 MPa and 5 s, respectively, the filling rate of micro-structured polymer can reach a maximum value of 99.30%.

## 4. Conclusions

The micro-grinding technology using a trued V-tip diamond grinding wheel is proposed to machine regular and accurate micro-grooved array structures on the surface of the mold core. The micro-structured polymers are efficiently and precisely fabricated using micro injection molding based on a micro-ground mold core. It may realize low-cost mass-production of micro-structured polymer components. The main results can be concluded as follows:

1. The highest form accuracy of the micro-ground mold core and filling rate of the micro-structured polymer can reach to 4.05 µm and 99.30%, respectively. The minimum angle relative error of the micro-formed polymer is only 0.4%.
2. The surface roughness *R*_a_ of micro-formed polymer side can be maintained below 0.1 μm. The least *R*_a_ of micro-ground mold core side is 0.271 μm, and the corresponding *R*_a_ of micro-formed polymer can also reach a minimum value of 0.052 μm.
3. The form accuracy of micro-ground mold core positively influences the filling rate of micro-formed polymer. The filling rate of micro-structured polymer basically increases with the increasing machining accuracy of the mold core.
4. The injection pressure has the greatest influence on the filling rate of micro-formed polymer, while the melt temperature has the least influence.

## Figures and Tables

**Table 1:** Micro-grinding conditions of mold core using a V-tip diamond grinding wheel.

CNC Grinder	SMART B818 III
Diamond grinding wheel	SD3000, resin bond, diameter *D* = 150 mm, width *B* = 4 mm, Wheel speed *N* = 3000 r/min
Workpiece	Ti_3_SiC_2_ ceramic mold core
Rough machining	Feed speed *v*_f_ = 1000 mm/min, depth of cut *a* = 5 µm
Finish machining	Feed speed *v*_f_ = 100 mm/min, depth of cut *a* = 1, Σ*a* = 10 µm
Coolant	Emulsion

**Table 2:** The designed V-grooved structure parameters of mold cores.

Sample	V-Groove Angle *α* (°)	V-Groove Depth *h* (µm)	V-Groove Space *b* (µm)
A_m_	90	100	400
B_m_	90	100	500
C_m_	90	100	600
D_m_	90	150	400
E_m_	90	150	500
F_m_	90	150	600

**Table 3:** Experimental parameter lists of micro injection molding.

No.	Melt Temperature *T* (°C)	Injection Speed *v* (mm/s)	Injection Pressure *P* (MPa)	Holding Time *t* (s)
1	200	40	7	3
2	205	40	7	3
3	210	40	7	3
4	215	40	7	3
5	220	40	7	3
6	210	30	7	3
7	210	35	7	3
8	210	45	7	3
9	210	50	7	3
10	210	40	5	3
11	210	40	6	3
12	210	40	8	3
13	210	40	9	3
14	210	40	7	1
15	210	40	7	2
16	210	40	7	4
17	210	40	7	5

**Table 4:** The V-grooved structure parameters of micro-ground mold cores.

Sample	V-Groove Angle *α*_1_ (°)	V-Groove Depth *h*_1_ (µm)	V-Groove Space *b*_1_ (µm)
A_m_	90.88	99.72	400.43
B_m_	91.87	106.64	503.17
C_m_	91.80	100.37	604.17
D_m_	91.17	148.10	401.97
E_m_	91.19	152.61	506.52
F_m_	91.35	153.93	599.86

**Table 5:** The V-grooved structure parameters of micro-structured polymers.

Sample	V-Groove Angle *α*_2_ (°)	V-Groove Depth *h*_2_ (µm)	V-Groove Space *b*_2_ (µm)
A_w_	89.98	98.74	400.40
B_w_	89.98	106.50	500.40
C_w_	89.12	98.43	600.50
D_w_	90.81	147.15	398.00
E_w_	89.26	150.77	501.40
F_w_	90.26	151.55	598.00
